# Effects of a *Scutellaria baicalensis*/*Crataegus laevigata*, magnesium and chromium supplement on stressed individuals: A randomised, double-blind, placebo-controlled, crossover trial

**DOI:** 10.1177/02698811251381261

**Published:** 2025-11-05

**Authors:** Fiona Dodd, Ramon Weishaupt, Philipp K. M. Katumba, Rian Elcoate, Emma Wightman

**Affiliations:** 1 Brain, Performance and Nutrition Research Centre, School of Psychology, Northumbria University, Newcastle upon Tyne, UK; 2 Medical Department, A.Vogel AG, Roggwil, Switzerland; 3 SIHLMED Integrative Medicine, Zürich, Switzerland; 4 Nutrition Trials At Northumbria (NUTRAN), Northumbria University, Newcastle upon Tyne, UK

**Keywords:** herbal supplement, *Scutellaria baicalensis*, Chinese Skullcap, *Crataegus laevigata*, hawthorn, well-being, cognition, sleep

## Abstract

**Background:**

Chronic stress is prevalent in most societies, impairing cognition, mood, and social functioning. Research suggests that supplements containing extracts from *Scutellaria baicalensis* root and *Crataegus laevigata* fruits may offer support in this regard.

**Aims:**

To investigate the acute and chronic effects of a *S. baicalensis*, *C. laevigata*, and magnesium/chromium containing herbal supplement on psychological well-being, cognition, and sleep in subjectively stressed but principally healthy adults.

**Methods:**

Forty-three participants (35 analysed) aged 18–75 years received the herbal supplement and a placebo for 15 days. Psychological well-being, and sleep were measured after 7 and 15 days of treatment. Cognitive performance was evaluated following a bolus dose of two tablets and after 15 days, with and without an observed multitasking stressor.

**Results:**

The herbal supplement significantly improved performance on a task of attention and working memory (as evidenced by a reduction in serial 3’s subtraction errors) following an acute dose and improved working memory performance (an increase in the number of correct serial 7’s subtraction) during the stressor, irrespective of dose. Cognitive effects were less consistent in the absence of the stressor. Chronic supplementation improved mood and anxiety, reducing total mood disturbance, anger/hostility, and Trait anxiety scores. A higher proportion of subjects experienced ⩾30% gains in social satisfaction scores after 7 days. No serious adverse effects were reported.

**Conclusions:**

The herbal supplement is safe and enhances mood, reduces subjective anxiety, and improves cognition under stress, though cognitive effects are variable without stress exposure.

The study was registered on clinicaltrials.gov (identifier: NCT05757050).

## Introduction

The relationships between stress, sleep, and cognition are complicated but offer great potential to human health and well-being if disentangled. Recent global reports underscore the pervasive nature of stress, with its prevalence rising across multiple sectors. For instance, Kuehnl et al. (2019) found that 22% of European workers suffer from work-related stress, while Stallman (2010) reported that 19.2% of surveyed students experienced mental health problems, with an additional 67.4% reporting subsyndromal symptoms, with women being particularly impacted. The relationship between stress and sleep is recognised as being bidirectional (Goldstein and Walker, 2014; Meerlo et al., 2008). During the COVID-19 pandemic, sleep problems were reported by 40% of respondents, suggesting a significant intersection between stress and sleep (Jahrami et al., 2021), and serving as an example of how stress can amplify sleep disturbances (Ballesio et al., 2022; Chen and Cheng, 2024; Mandelkorn et al., 2021).

Constant stress increases the risk for a high number of serious diseases, such as cardiovascular diseases (Kivimäki and Steptoe, 2018). Acute, high distress has been shown to negatively impact cognitive performance, particularly memory, attention, and decision-making via the hypothalamus–pituitary adrenal axis. Research suggests that stress-related hormonal and neural changes can interrupt normal cognitive processes, reducing mental efficiency (Hinds and Sanchez, 2022; Merz and Wolf, 2022).

The mechanisms underpinning these effects of stress include disruption of serotonin (Chaouloff, 2000), dopamine (Cabib and Puglisi-Allegra, 2012; Pruessner et al., 2004), and noradrenaline pathways (Reyes and Chandler, 2023), which are all critical for maintaining mood, motivation, and cognitive performance. This neurotransmitter dysregulation impairs concentration and executive function, increasing susceptibility to depression and underscoring the therapeutic potential of targeted pharmacological interventions (Rihmer et al., 2022; Yan and Rein, 2022).

Stress and anxiety are inextricably linked to cognitive function, and a recent review confirmed that sleep deprivation evinces clear negative effects on functions like short-term memory, attention, processing speed, and vigilance. Conversely, extended sleep was found to improve overall cognition, as compared to baseline (Mantua and Simonelli, 2019). As above, the role of sleep in cognitive functions is believed to be largely restorative; Xie et al. (2013), for example, argue that the relative inactivity of the brain during sleep affords a period where waste by-products (e.g. in the building and deamination of neurotransmitters), which have accrued during waking, can be removed from the central nervous system. Equally, this ‘down-time’ is also widely believed to facilitate the learning process by reprocessing memory formations from the wake period (Stickgold, 2005); potentially underpinned by cholinergic changes in the hippocampus during rapid eye movement sleep (Graves et al., 2001); which supports the abovementioned correlations between sleep quality/quantity and cognitive task performance.

The herbal extracts and essential minerals within the supplement under investigation all, to a greater or lesser extent, impact upon stress, sleep, and cognition, via interaction with several physiological mechanisms. *Scutellaria baicalensis* (also known as Chinese skullcap) root extracts and constituents, for example, are able to interact with the gamma-aminobutyric acid (GABA) and dopaminergic neurotransmitter system (Hui et al., 2002; Limanaqi et al., 2020) and whilst no randomised intervention trials in humans have observed outcomes here, rodent studies suggest that *S. baicalensis* impacts positively on sleep activity (Chang et al., 2011), mood and anxiety (Limanaqi et al., 2020; Ma et al., 2024). *Crataegus laevigata* (also known as hawthorn) too has been observed to exert positive effects on sleep, specifically insomnia, as well as anxiety and other markers of health, which may be due to their active phenolic components, specifically chlorogenic acid (see Nazhand et al., 2020, for review). Indeed, in our own lab recently, we have seen positive cognitive outcomes following supplementation with chlorogenic acid in humans (Jackson et al., 2020, 2021a). Magnesium, an essential mineral and cofactor within the body (Swaminathan, 2003), has been shown to benefit populations with a vulnerability to anxiety (Boyle et al., 2017; Rawji et al., 2024) and those experiencing sleep disturbances (Arab et al., 2023; Rawji et al., 2024). Chromium, an essential trace mineral important for carbohydrate and lipid metabolism, has been shown to improve memory function in older adults with early memory decline (Krikorian et al., 2010) and has demonstrated antidepressant activity in both animal and human models (Młyniec et al., 2014), possibly through modulation of serotonergic activity (Di Lisi et al., 2025).

Taken together, research to date strongly suggests the investigation of these interventions in terms of potential effects on stress, cognition, sleep, and well-being in a principally healthy human sample and, to ensure likely impact here, it seems axiomatic to investigate these effects in those who have a subjective appraisal of relatively high stress. Therefore, the aim of the present study was to investigate the acute and chronic effects of supplementation with an active herbal intervention containing *S. baicalensis* root and *C. laevigata* fruit extracts in a combination with magnesium/chromium on measures of well-being, mood, sleep, stress, and cognition in healthy human volunteers who subjectively report as stressed.

## Methods

### Study design

A randomised, placebo-controlled, double-blind, crossover design was utilised. Participants attended the Brain, Performance, Nutrition Research Centre (BPNRC) laboratory at Northumbria University and were assessed after 15 days of supplementation with a herbal supplement, and a matched placebo. Treatment order was counterbalanced and separated by a 14-day washout period. The study was performed in accordance with the ethical principles that have their origin in the Declaration of Helsinki (1996). The trial was conducted in compliance with protocol/Good Clinical Practice/applicable regulatory requirements and commenced only when a favourable ethical opinion was obtained from the University of Northumbria Department of Psychology Ethics Committee, UK (approval number 1942). CONSORT reporting guidelines were also used (Schulz et al., 2010).

### Determination of sample size

A power calculation using G*Power (Faul et al., 2007) was made with reference to a small effect size (Cohen’s *f* = 0.2) and a minimum power of 0.8. Based on these variables, a total sample size of 36 participants was estimated for a two-group, repeated measures, within-factors design.

### Study population

A total of 43 healthy, male and female adults were randomised, of which five withdrew, and three further participants were withdrawn due to major protocol violation. Of these three, two were withdrawn due to poor treatment compliance, and one participant was withdrawn due to medication use. The remaining 35 participants (18 female/17 male) aged 18–75 years (mean = 47.14, standard deviation (SD) = 18.48) self-reported as experiencing stress and scored ⩾13 on the Perceived Stress Scale (PSS) but were otherwise healthy. Participants reported being free from any relevant medical condition or disease, including psychiatric and neurodevelopmental disorders, that would impact the study outcomes. Blood pressure (BP) and body mass index (BMI) were measured at screening, and participants were enrolled into the study if their BP measured <159 mmHg systolic and <99 mmHg diastolic and their BMI was within the range 18.5–35 kg/m^2^. Participants confirmed they were not currently taking any relevant pharmaceuticals and had not taken any antibiotics within 4 weeks of screening. They also confirmed they had not taken part in another clinical trial or nutrition intervention study within the past 30 days. A full list of the inclusion and exclusion criteria can be found in Supplemental File 1. Written, informed consent was obtained from participants prior to any research-related procedures being performed. Participants were recruited via an opportunity sample from Northumbria University students and staff and the general population.

### Treatment

Participants received the active herbal supplement as a chewable tablet containing *S. baicalensis* (400 mg) and *C. laevigata* (40 mg), Magnesium (56.3 mg), and Chromium (20 µg) per tablet, or a matched placebo in a counterbalanced order. The interventions were provided in chewable tablet form and were matched in appearance and taste with a natural citrus flavour. The full composition of the active herbal supplement and placebo is listed in Supplemental File 2. The interventions were manufactured by A.Vogel AG (Roggwil, Switzerland) and supplied in boxes labelled as verum and placebo.

A fully counterbalanced computer-generated randomisation schedule was created (www.randomization.com), grouped by age and gender, and treatment codes A and B were assigned to treatment in blocks of 10. Bottles (2 per treatment period) were labelled according to the counterbalancing schedule for treatment periods 1 and 2 by an independent third party who had no further involvement with the trial procedures. All interventions were labelled according to the requirements of local law and legislation. Participants were allocated to a randomisation code by the researcher at the first testing visit; codes were allocated sequentially based on the age and sex of the participant. Participants who discontinued post-randomisation were replaced using the same treatment order. Participants were supplied with two bottles, containing 28 tablets each, to cover each 15-day treatment period. Participants were instructed to consume two tablets per day whilst at home: one in the morning, and one in the afternoon. The morning dose was instructed to be consumed at least 1 hour after breakfast, and the afternoon dose at least 1 hour before their evening meal. Participants were instructed not to consume their usual treatment when they returned to the lab on day 15, as they would receive their daily dose as part of the testing visit. On testing visits, treatment was consumed as one bolus dose of two tablets. There was a minimum of 14 days between the last dose of the first investigational product and the first dose of the second. This was the ‘washout period’.

### Participant-reported outcome measures

These outcome measures were completed once per testing visit and once during the interim assessment.

#### Subjective Sleep via Consensus Sleep Diary

This is an established 12-item measure of subjective sleep continuity (Carney et al., 2012). The sleep diary was completed in the morning and the following variables were created from the diary; Sleep latency (how long, in minutes, the individual felt it took them to fall asleep after intending to sleep); Time in bed (how long, in minutes, the individual reports being in bed intending to sleep); Number of awakenings (number of perceived awakenings over the sleep period); Wake after sleep onset (how long, in minutes, the individual reports being awake during the night after sleep initiation); Total sleep time (how long, in minutes, the individual reports being asleep during the night between initiation and termination of sleep, accounting for nocturnal wake periods); Sleep efficiency (total sleep time divided by time in bed × 100, expressed as a percentage).

As a measure of sleep quality, four items (each scored on a 0–4 scale) covering nocturnal physical and psychological tension, sleep enjoyment, and feelings of restedness were assessed. Items were analysed individually and summed (range 0–16), with responses to ‘sleep enjoyment’ and ‘feelings of restedness’ reversed, so that a higher score indicated a negative response on these items. When summed, higher overall scores indicated poorer overall sleep quality.

#### Patient-Reported Outcomes Measurement Information System Sleep Disturbance

An eight-item measure of sleep disturbance (Yu et al., 2011). Each item was rated (on a scale from 1–5) and summed to create a single value (range 8–40) with higher scores indicating higher levels of sleep disturbance.

#### Perceived Stress Scale

The (PSS) is a 10-item measure of perceived stress that assesses the degree to which situations in one’s life are appraised as stressful (Cohen et al., 1983). In the present study, participants were asked to reflect on the previous week in relation to their responses. Each item is rated using a five-point scale (0–4) and summed to create a single value (range 0–40), with higher scores indicating higher levels of perceived stress.

#### State Trait Anxiety Inventory

This is a 40-item measure of current and general anxiety levels (Spielberger et al., 1983). Each item is rated on a scale from 1 to 4. Scoring creates two components: state (current) anxiety (20 items) and Trait (general) anxiety (20 items), with a range for each between 20 and 80. Following transformation through reversed coding, higher scores indicate higher levels of anxiety.

#### Depression Anxiety and Stress Scale

This is a 21-item measure of mood over the previous week, with each item rated on a scale from 0 to 3 (Lovibond and Lovibond, 1995). Scoring creates three component scores; depression, anxiety, and stress (each on a scale of 0–21) with higher scores indicating higher symptomology. Total scores can be derived by multiplying the sum of all three component scores by 2.

#### World Health Organization Quality of Life Questionnaire-Bref

The World Health Organization Quality of Life Questionnaire-Bref (WHOQOL-Bref) includes 26 questions, which includes an item relating to each of the 24 facets included in the WHOQOL-100, along with two items from the overall quality of Life and General Health facet. The assessed domains comprise physical health, psychological health, social relationships, and environment, and participants were asked to respond in relation to the past week. Scores are converted to a 0–100 scale as per published guidelines (WHO, 1996) with higher scores indicating more positive outcomes for each of the outcome measures.

#### Profile of Mood States

The Profile of Mood States (POMS) is a 35-item measure of current mood, assessed over the past week (Heuchert and McNair, 2012), where each item is rated on a scale from 0 to 4 and can be consolidated into Anger-Hostility, Confusion-Bewilderment; Depression-Dejection; Fatigue-Inertia; Tension-Anxiety; Vigour-Activity, and Friendliness are also calculated (automated online by publisher). To determine current negative mood (Total Mood Disturbance), both negative and positive items are summed, and then positive scores are subtracted from the negative score. Outcome measures are *T*-scores, with higher scores on Total Mood Disturbance indicative of negative mood states, and lower scores on positive mood states potentially indicating a problem.

#### Stress Visual Analogue Scales

Participants also rated their feelings in relation to the following four antonyms, anchored by ‘not at all’ and ‘extremely’; Relaxed; Stress; Anxious; Calm. Each line was scored as a % along the line on a 0–100 scale, with higher scores indicating higher levels of each outcome.

#### Visual Analogue Mood Scales

These mood scales were a set of 18 antonyms where participants moved a marker along a line to describe how they currently feel. Each line was scored as a percentage along the line on a 0–100 scale. The factors were labelled Alertness (11 items: Alert-inattentive; lethargic-energetic; clumsy-coordinated; lively-sluggish; quick-witted-slow-witted; sharp-dull; exhausted-refreshed; bored-engaged; focused-unfocused; drowsy-awake; motivated-unmotivated), Stress (four items: tense-relaxed; fearful-fearless; stressed-carefree; peaceful-troubled), and Tranquillity (three items: tranquil-agitated; contented-discontented; friendly-hostile). The individual item scores were combined to give an average (% along the line), with higher scores indicating higher levels of each factor.

### Cognitive measures

The Computerised Mental Performance Assessment System (COMPASS) cognitive measures were completed twice per testing visit.

**Figure 1.**
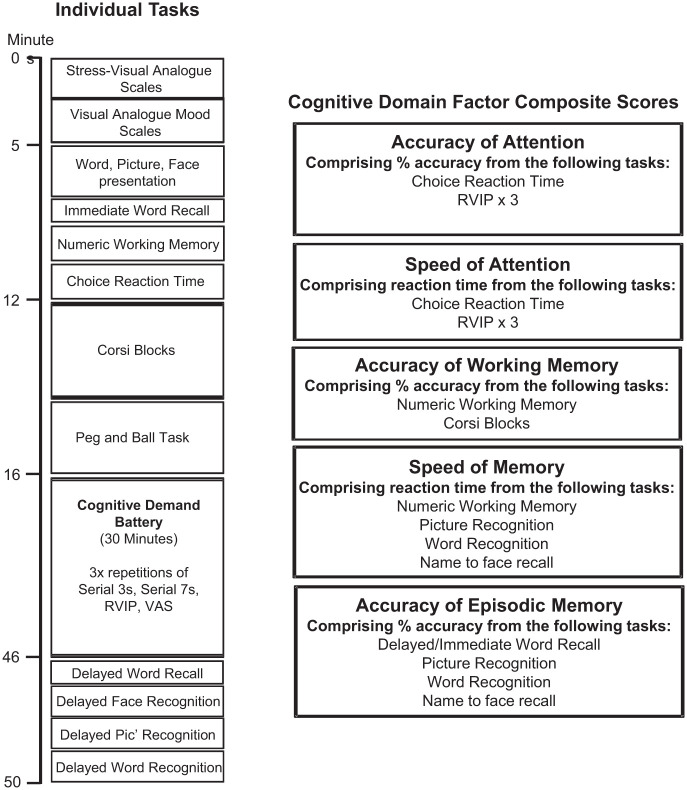
COMPASS individual tasks and cognitive domain factors. COMPASS: Computerised Mental Performance Assessment System.

The COMPASS testing system delivers a bespoke collection of tasks, with fully randomised parallel versions of each task delivered at each assessment for each individual. The battery has been in use within the BPNRC for over 15 years and is currently in use within a number of United Kingdom, United States, New Zealand, and Australian Universities, companies and research organisations. In the face of little evidence of domain-specific cognitive effects of the investigational product, this paradigm was somewhat exploratory, with the cognitive tasks used herein assessing multiple facets of cognition. The individual tasks included numeric working memory; choice reaction time; Corsi blocks; peg and ball; immediate word recall; delayed word recall; name to face recall; delayed word recognition; picture recognition. The order in which they were presented, the approximate timings, as well as the collapsible cognitive domain composite factors are represented in Figure 1. A full description of all tasks is provided in Supplemental File 3.

#### Cognitive domain composite factor scores

Cognitive domain factor scores were derived by collapsing the data from individual tasks into ‘composite scores’ relating to broad cognitive domains, with the purpose of minimising the impact of the variability in individual test scores (see Figure 1). Similar factors have been used previously and have also been shown to be sensitive to nutritional manipulation (Dodd et al., 2024; Kennedy et al., 2019; Patan et al., 2021). Prior to calculation, each relevant task outcome for each intervention at each assessment period was converted to a *Z*-score. The cognitive domain composite factor scores were selected as the primary exploratory outcome measure of this trial.

#### Cognitive demand battery

The objective of this battery was to assess the impact of treatment on speed/accuracy and mental fatigue during continuous performance of cognitively demanding tasks. Here, participants completed the ~10-minute battery of tasks (serial 3’s (2 minutes), serial 7’s (2 minutes), rapid visual information processing (RVIP) (5 minutes) and a mental fatigue scale (~1 minute), 3 times in immediate succession (i.e. for a continuous period of 30 minutes). Application of this battery has been shown to reliably increase self-ratings of ‘mental fatigue’ and to be sensitive to a number of herbal and natural interventions (Kennedy et al., 2008; Reay et al., 2005, 2006). A full description of the cognitive demand battery (CDB) and associated tasks is provided in Supplemental File 3.

### Observed multitasking stressor

The observed multitasking stressor (OMS) is a stressor paradigm designed specifically for repeated use. Recent trials have evidenced the validity of this method in inducing acute subjective and physiological stress in healthy adult volunteers and the alleviation of this via supplementation (Jackson et al., 2021a; Kennedy et al., 2020). The paradigm involves the completion of demanding cognitive tasks concurrently (4 minutes each of oral serial 3’s, serial 7’s and serial 17’s subtraction tasks), whilst being observed by a panel of two researchers, in addition to the participant being recorded by a video camera. During the procedure, saliva samples are taken at key time-points (prior to and following the stressor) in order to assess levels of cortisol and alpha amylase. The cognitive tasks are assessed for performance, and subjective stress is also measured before and after the procedure by the State Trait Anxiety Inventory (STAI)-State subscale. The OMS was completed twice per testing visit. Therefore, four assessments were captured within each treatment period. The multitasking element of the stressor comprised the concomitant performance of the following two tasks at the same time in three concurrent blocks of 4 minutes.

#### Serial subtractions

During each 4-minute block, participants were instructed to count out loud backwards in 3’s, 7’s, or 17’s (please see previous description of this task).

#### Tracking

**Figure 2.**
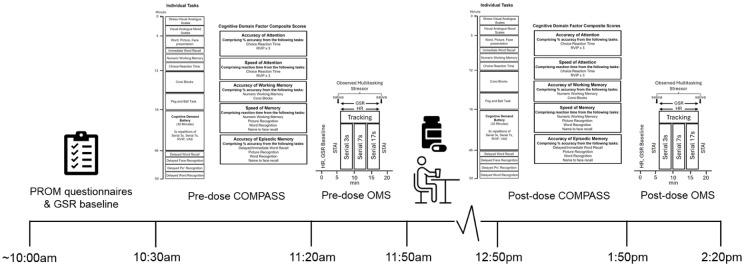
Acute and chronic testing visit procedure.

Whilst performing the three blocks of verbal serial subtraction tasks, participants also completed a computerised tracking task, which required the participants to use the mouse to move a cursor and track an asterisk as it moved across the screen. On screen, the asterisk moved at a rate of approximately 6 cm/s on a 35 cm laptop screen (~168 pixels/s) in a smooth random path. Participants were instructed to keep the cursor as close to the asterisk as possible. The distance between the target and the cursor was computed every 100 milliseconds, and the resulting data converted to an accuracy score representing the distance of the cursor from the asterisk in pixels, averaged across the 4-minute block of task performance (with a lower score indicating improved performance). A visual representation of the stressor assessment is displayed in Figure 2.

#### Heart rate and galvanic skin response

Heart rate (HR) and galvanic skin response (GSR) were measured upon arrival on each testing day and throughout performance of the stressor using the Vilistus Digital Sampling Unit (Durham Systems Management Limited, UK). GSR sensors, which measure relative changes in skin conductance, were attached to the middle and forth fingertips on the participant’s non-dominant hand using Velcro straps. The HR sensor clip, which measures blood volume pulse, was placed on the tip of the index finger or thumb on the non-dominant hand. These sensors were attached at least 1 minute prior to the commencement of recording to allow for stabilisation of the readings. The unit measures 32 and 128 samples per second for GSR and HR, respectively.

#### Salivary cortisol and salivary α-amylase

Saliva samples were collected using salivettes (Sarstedt Ltd, UK) upon arrival on each testing day and immediately before and after each OMS assessment to measure salivary cortisol and alpha amylase. Salivettes were weighed prior to and following sample collection in order to calculate the total sample volume. Samples were spun down at 1,000 *g* for 2 minutes then transferred into Eppendorfs and frozen at −80°C. Before assaying, the samples were thawed, and the cortisol and α-amylase levels in the saliva samples were measured using ELISA (Salimetrics Ltd, UK).

### Procedure

**Figure 3.**
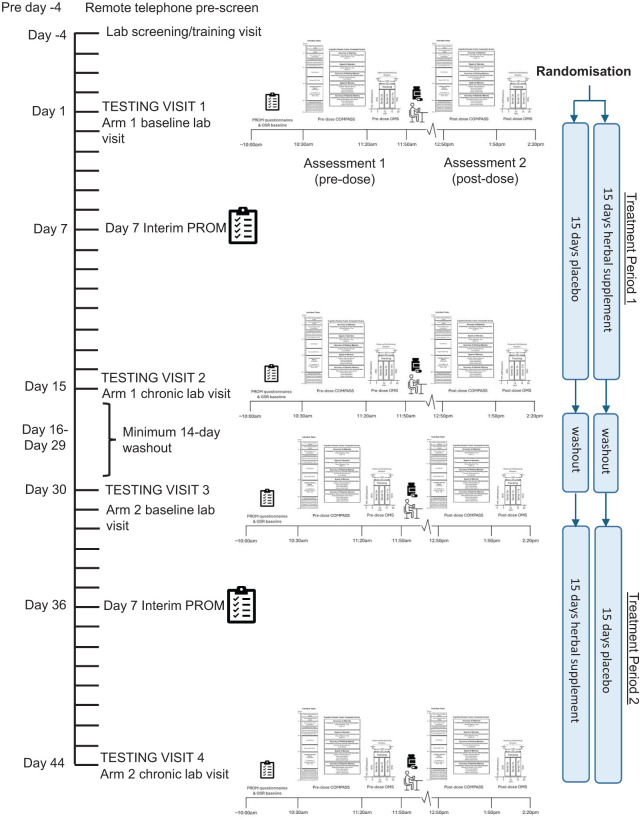
Timeline of study incorporating COMPASS and observed multitasking stressor assessments. COMPASS: computerised mental performance assessment system.

Participants initially completed a telephone pre-screen where they provided consent to participate and confirmed they met the study eligibility criteria, before attending the Brain, Performance and Nutrition Research Centre Laboratory (Northumbria University, UK) on five separate occasions. The first was an in-person screening and training visit where informed written consent was obtained and demographic and anthropometric measurements (height, weight, and BP) were taken. Participants were also trained on the cognitive tasks they would be required to complete during the testing visits. Participants attended the laboratory at a pre-arranged time in the morning on four separate occasions (visits 1–4) from 9 March 2023 to August 2023. The first and third visits comprised the baseline assessments. Visits 2 and 4 were chronic assessments and occurred 14 days (±2 days) after visits 1 and 3, respectively. Each visit was identical, except for the intervention consumed. See Figure 2 for a schematic depicting the procedure during a study day and Figure 3 for a schematic depicting the overall study timeline. Upon arrival at visits 1–4, participants were screened for continued eligibility. They then provided baseline GSR and HR readings (~5 minutes), a day-baseline saliva sample, and the participant reported outcome measures (PROMS) questionnaires. Following this, participants completed a baseline assessment of the COMPASS cognitive task battery (see Figure 1 for a schematic depicting the individual COMPASS tasks). After a short (~10 minutes) break, participants were taken to an ‘interview’ room where they underwent a baseline completion of the stressor for 20 minutes, in front of a panel of two observers, whilst also being video recorded and having their GSR and HR readings measured throughout. The STAI-State subscale was completed, and a saliva sample was provided in the lab immediately prior to and after the stressor. Participants were then provided with their daily treatment (2 × tablets; bolus dose), followed by a standardised lunch. The second (i.e. the post-dose) assessment started after an hour-long absorption period, and here participants completed the COMPASS cognitive assessment, followed by the stressor. Before leaving on visits 1 and 3, participants were provided with their treatment and instructed to take one in the morning (at least an hour after breakfast) and one in the afternoon, (at least 1 hour before their evening meal). Participants were also instructed to complete an interim PROMS assessment on day 7 (±2) in each treatment period. A full description of the procedure is provided in Supplemental File 4.

### Statistical analysis

All primary and secondary outcomes were analysed using the per-protocol population. The per-protocol population included those participants who completed all study visits and procedures as directed and who did not have any major protocol violations. For the data collected during the study visits, the general statistical approach comprised the analysis of data collected following each treatment period in the presence and absence of an acute dose (i.e. visits 2 and 4), including data collected at the pre-intervention assessment (i.e. visits 1 and 3) as a covariate.

The MIXED procedure in SPSS (version 26.0, IBM Corp., Armonk, NY, USA) was used for all analyses. Multilevel modelling was used to fit the composite or individual score for each outcome measure. Intervention (herbal supplement, placebo) and Day (Day 1 (acute), Day 15 (pre-dose chronic), Day 15 (post-dose chronic)) were entered into the model as fixed effects along with their interaction and respective baseline score as a covariate for the COMPASS cognitive domain factor composite scores and OMS measures. Repetition (1–3) was included as an additional fixed effect for the analysis of the CDB outcome measures. Assessment (pre-OMS, post-OMS) was included as an additional fixed effect for the analysis of outcome measures captured before and after the psychosocial stressor (cortisol, a-amylase and STAI-state subscale). ‘Intervention’ (herbal supplement, placebo) and ‘Day’ (Day 7 (interim), Day 15 (chronic)) were entered into the model as fixed effects along with their interaction and respective baseline score as a covariate for the PROMS measures. For each model, restricted maximum likelihood estimation methods were used, and covariance matrix structure was chosen based on the structure that produced the lowest Schwarz’s Bayesian Criterion; an indication of the best fitting model (Drton and Plummer, 2017). ‘Participant’ was entered as a random effect where appropriate. ‘Age’ was entered into the model as a covariate if it improved model fit. Significant interaction effects were analysed further using pairwise comparisons and adjusted for multiple comparisons according to Šidák (1967). Given the absence of sufficient evidence for domain-specific cognitive effects of the investigational product, the study was designed to assess multiple facets of cognition (and psychological well-being). The primary outcome measure therefore constituted factors derived from the cognitive domain composite scores (accuracy of attention, speed of attention, speed of memory, accuracy of working memory, and accuracy of episodic memory). An additional Chi^2^ test was conducted as an exploratory analysis on PROMS to assess the proportion of participants per arm achieving ⩾30% improvement in each outcome measure at each time point.

## Results

**Figure 4.**
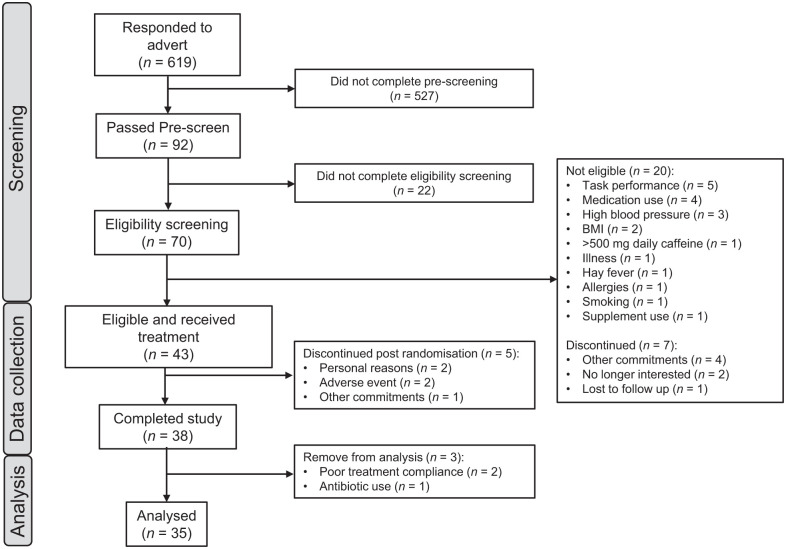
Flow diagram of disposition of subjects throughout the study.

Forty-three participants were randomised to receive treatment. Five participants withdrew post-randomisation following testing visit 1. A further three participants were removed from the per-protocol analysis due to major protocol violations, therefore, 35 datasets were eligible for analysis. See Figure 4 for the complete participant disposition diagram, Supplemental File 5 for the outcomes from the main mixed models analyses, and Supplemental File 6 for all raw data. Only those outcomes where a significant effect of treatment was observed are reported.

### Demographic and other baseline characteristics

Participant demographics and baseline characteristics are summarised in Tables 1 and 2.

**Table 1.** Baseline characteristics (*N* = 35).

Measure	*N* = 35	
	Mean	SD
Age (years)	47.14	18.48
Education (years)	16.60	2.94
Caffeine consumption (mg/day)	145.84	110.34
Alcohol consumption (units/week)	3.58	5.00
Systolic BP (mm/Hg)	123.59	14.89
Diastolic BP (mm/Hg)	79.59	8.73
Heart rate (beats per minute)	70.73	9.22
Weight (kg)	72.31	11.62
Height (cm)	168.57	10.71
BMI (kg/m^2^)	25.39	2.90
Baseline PSS score	18.24	5.17
Baseline trait score	43.55	10.71

Caffeine consumption in mg/day; low consumption = 0–120 mg/day, moderate = 120–400 mg/day, high = >400 mg/day (Addicott et al., 2009).

BMI: body mass index; BP, blood pressure; PSS: Perceived Stress Scale; SD: standard deviation.

**Table 2.** Demographic baseline characteristics (*N* = 35).

Measure	*N* = 35	
	Count	%
Race (self-described)		
African	1	2.9
Bangladeshi	1	2.9
British	4	11.4
Chinese	1	2.9
Greek	1	2.9
Indian	2	5.7
Mixed	1	2.9
White	8	22.8
White British	16	45.7
Wear glasses (yes/no)	16/19	45.7/54.3
Handedness (left/right)	2/33	5.7/94.3
Highest level of education		
Level 1-5 (NVQ level 1-5, BTEC Intro-5, GCSE A*-G, AS & A-levels, HND, HNC, Dip HE/FE, Found. Deg)	11	31.4
Level 6 (Bachelor’s Degree, GradDip, Grad Cert., BTEC 6)	13	37.2
Level 7–8 (Doctorate, Masters, PGDip, PG Cert, BTEC 7)	11	31.4

### Measurements of treatment compliance and treatment guess

Compliance was based on returned tablet counts at the end of each intervention period. Participants who achieved <80% across one or both intervention periods, or who did not adhere to the treatment schedule were excluded from the per-protocol analysis. For participants entered into the per-protocol analysis, mean compliance during the placebo phase was 108.4% (SD = 14.7%). Mean compliance during the herbal supplement phase was 98.8% (SD = 7.5%). Overall (both treatment periods), mean treatment compliance was 103.6% (SD = 8.6%). Success of blinding was confirmed via treatment guess taken after each treatment period. Chi-squared analysis confirmed that participants were not able to correctly identify whether they had received the herbal supplement or placebo following either treatment period; treatment period 1, *p* = 0.247; treatment period 2, *p* = 0.352.

### Baseline comparisons

Pre-intervention data at testing visits 1 and 3 were analysed for effects across treatment groups to confirm an absence of baseline differences between the groups at each visit.

The only baseline differences observed across measures were a significant effect for Choice Reaction Time ‘CRT’ % correct (*F* (1, 34) = 6.112, *p* = 0.019), where at visit 1 baseline assessment, participants assigned to placebo had significantly higher Choice Reaction Time accuracy (98.88) than the herbal supplement (97.16) before treatment commenced.

### Participant-reported outcome measures

#### Trait anxiety

**Figure 5.**
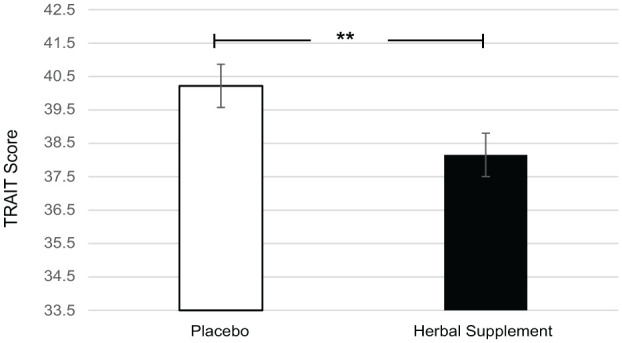
Estimated marginal means and standard error derived from the linear mixed model analysis for Trait anxiety. Significant treatment effect of the herbal supplement versus placebo. **p < 0.01.

A significant main effect of treatment was observed for anxiety on the Trait subscale of the STAI (*F* (1, 49.13) = 7.90, *p* = 0.007) where, irrespective of interim or chronic dose, participants reported significantly lower anxiety following the herbal supplement (38.15) as compared to placebo (40.22) (Figure 5).

#### Profile of Mood States

**Figure 6.**
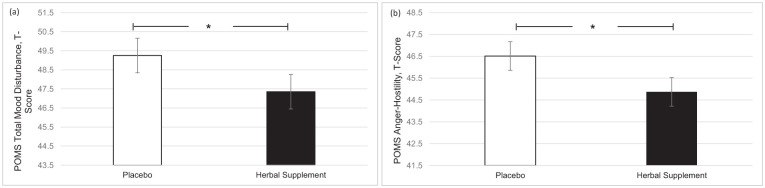
Estimated marginal means and standard error derived from the linear mixed model analysis for Profile of Mood States. Significant treatment effect of the herbal supplement versus placebo for (a) total mood disturbance and (b) anger-hostility. *p < 0.05.

A significant main effect of treatment was observed for total mood disturbance on the POMS questionnaire (*F* (1, 49.44) = 4.86, *p* = 0.032). Participants reported significantly lower total mood disturbance following the herbal supplement (47.35) as compared to placebo (49.25), irrespective of interim or chronic dose (Figure 6(a)). A significant main effect of treatment was also observed for anger-hostility (*F* (1, 48.21) = 5.86, *p* = 0.019), with participants reporting significantly lower anger-hostility following the herbal supplement (44.87) as compared to placebo (46.51), irrespective of interim or chronic dose (Figure 6(b)).

### World Health Organisation Quality of Life Questionnaire-Bref

The additional exploratory analysis indicated higher rates of social satisfaction in the herbal supplement group, with significantly more participants achieving ⩾30% increases in WHO-QOL social relationships scores at 7 days (6/34 vs. 1/35, χ² test, *p* = 0.042). This persisted as a trend at 15 days (6/34 vs. 2/34, *p* = 0.12).

### Subjective sleep diary

**Figure 7.**
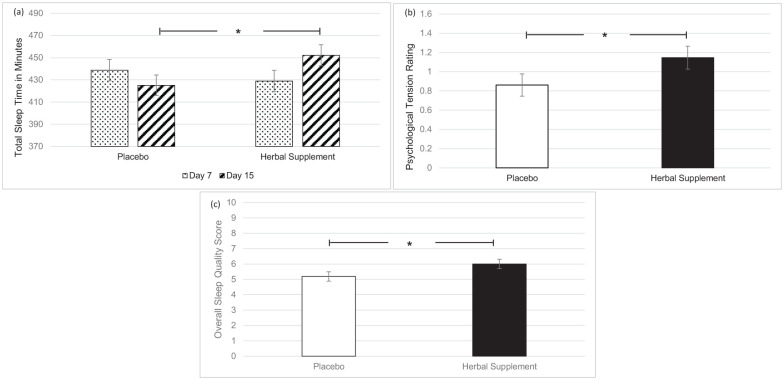
Estimated marginal means and standard error derived from the linear mixed model analysis for subjective sleep. Significant treatment effect of the herbal supplement versus placebo for (a) total sleep time, (b) psychological tension, and (c) overall sleep quality. *p < 0.05.

A significant Treatment × Day interaction was observed for total sleep time (*F* (1, 91.68) = 4.52, *p* = 0.036), with participants reporting significantly increased total sleep time following a chronic dose of the herbal supplement (452.16 minutes) as compared to placebo (425.18 minutes), on day 15 (*p* = 0.037) (Figure 7(a)). There was also a significant main effect of treatment for ratings of psychological tension in relation to sleep (*F* (1, 44.27) = 4.66, *p* = 0.036). Here, participants reported a significant reduction in psychological tension following placebo (0.86) as compared to the herbal supplement (1.15), overall and irrespective of interim or chronic dose (Figure 7(b)). A significant main effect of treatment for overall sleep quality was also observed (*F* (1, 37.96) = 5.26, *p* = 0.027). Participants reported significantly better sleep quality following placebo (5.19) as compared to the herbal supplement (6.00), overall and irrespective of interim or chronic dose (Figure 7(c)).

### Individual cognitive task performance

**Figure 8.**
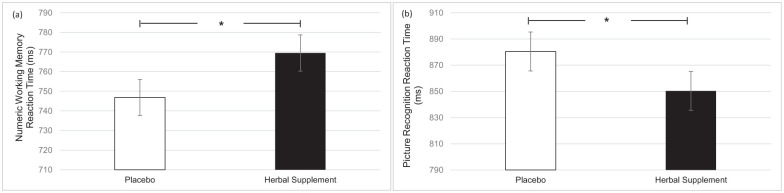
Estimated marginal means and standard error derived from the linear mixed model analysis for numeric working memory reaction time performance (a) and picture recognition reaction time performance (b). Significant treatment effect of the herbal supplement versus placebo. *p < 0.05.

A significant main effect of treatment was observed for numeric working memory reaction time (*F* (1, 45.56) = 4.67, *p* = 0.036). A significant reduction in numeric working memory reaction time was observed following placebo (746.82) as compared to the herbal supplement (769.50), overall and irrespective of dose (Figure 8(a)). A significant main effect of treatment was also observed for picture recognition reaction time (*F* (1, 60.98) = 5.21, *p* = 0.026). A significant reduction in picture recognition reaction time was observed following the herbal supplement (850.33) as compared to placebo (880.44), overall and irrespective of dose (Figure 8(b)).

### CDB and serial 3’s and 7’s subtractions

**Figure 9.**
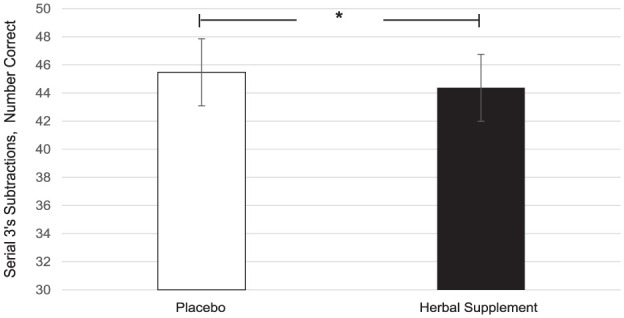
Estimated marginal means and standard error derived from the linear mixed model analysis for serial 3’s subtractions correct performance during the cognitive demand battery. Significant treatment effect of the herbal supplement versus placebo. *p < 0.05.

A significant main effect of treatment was observed for correct number of serial 3’s subtractions (*F* (1, 151.45) = 4.42, *p* = 0.037). A significant increase in the number of serial 3’s correct responses was observed following placebo (45.48) as compared to the herbal supplement (44.37) overall and irrespective of acute or chronic dose (Figure 9).

**Figure 10.**
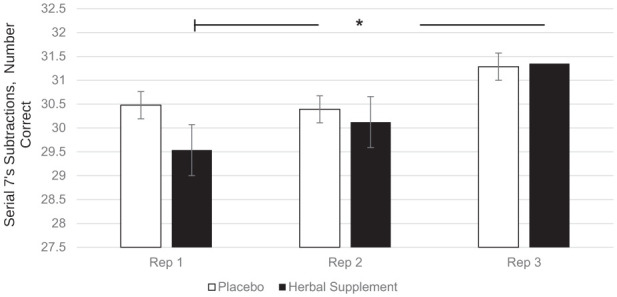
Estimated marginal means and standard error derived from the linear mixed model analysis for serial 7’s subtractions correct performance during the cognitive demand battery. Significant within-treatment effect. *p < 0.05.

A significant Within-Treatment × Repetition interaction was observed for the number of serial 7’s subtractions correct (*F* (2, 415.66) = 3.17, *p* = 0.043). Following the herbal supplement, performance improved across repetitions in the number of correct serial 7’s subtractions, with a significant increase in correct responses at repetition 3 (31.35), compared to repetition 1 (29.53), (*p* = 0.048), irrespective of acute or chronic dose (Figure 10).

### CDB and RVIP

**Figure 11.**
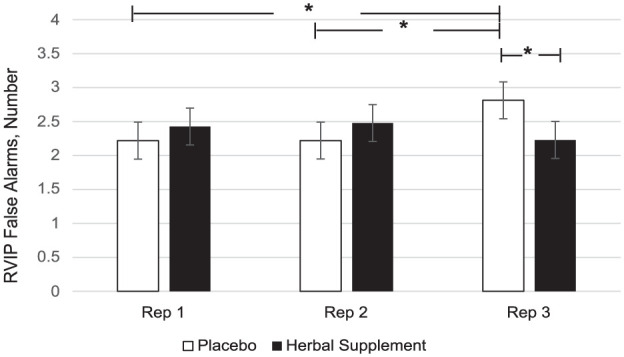
Estimated marginal means and standard error derived from the linear mixed model analysis for rapid visual information processing false alarms performance during the cognitive demand battery. Significant treatment effect of the herbal supplement versus placebo. Significant within treatment effects. *p < 0.05.

A significant Treatment × Repetition interaction was observed for RVIP false alarms (*F* (2, 403.63) = 4.41, *p* = 0.013). Here, a significant reduction in the number of false alarms was observed at repetition 3 following the herbal supplement (2.23) as compared to placebo (2.81) (*p* *=* 0.013) (Figure 11). A within-treatment effect was also observed. Following placebo, RVIP false alarms significantly increased at repetition 3 (2.81) as compared to repetition 1 (2.22), (*p* = 0.026) and repetition 2 (2.22), (*p* = 0.023). In contrast, this pattern was not observed following the herbal supplement (Figure 11).

### OMS and serial 3’s and 7’s subtractions

**Figure 12.**
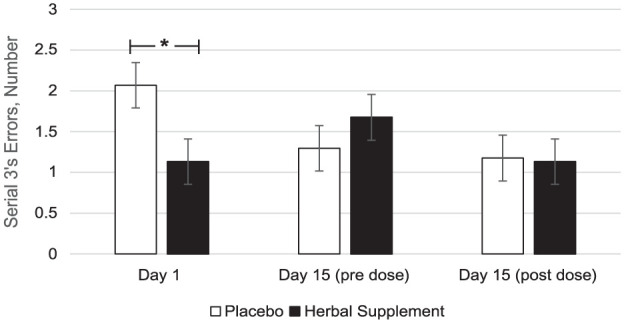
Estimated marginal means and standard error derived from the linear mixed model analysis for serial 3’s subtraction errors during the observed multitasking stressor. Significant treatment effect of the herbal supplement versus placebo. *p < 0.05.

A significant Treatment × Day interaction was observed for serial 3’s errors during the OMS (*F* (2, 127.09) = 3.49, *p* = 0.033), with a significant decrease in serial 3’s errors following acute dose of the herbal supplement (1.13) compared to placebo (2.07) (*p* = 0.015) (Figure 12).

**Figure 13.**
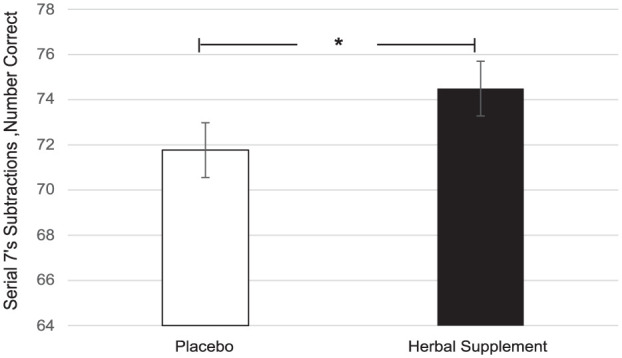
Estimated marginal means and standard error derived from the linear mixed model analysis for serial 7’s subtractions correct performance during the observed multitasking stressor. Significant treatment effect of the herbal supplement versus placebo. *p < 0.05.

A significant main effect of treatment was observed for serial 7’s correct during the OMS (*F* (1, 59.95) = 4.14, *p* = 0.046). Here, there was a significant increase in the number of serial 7’s correct during the stressor following the herbal supplement (74.49) as compared to placebo (71.77), overall and irrespective of acute or chronic dose (Figure 13).

### OMS and HR

**Figure 14.**
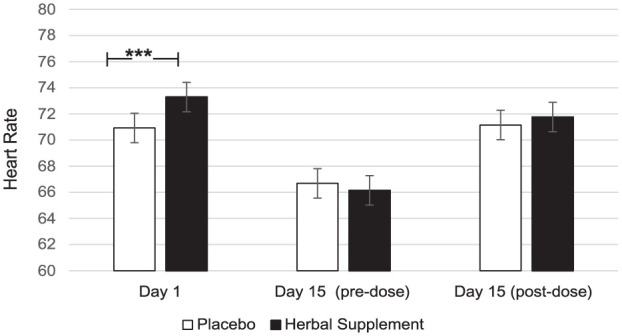
Estimated marginal means and standard error derived from the linear mixed model analysis for heart rate during the observed multitasking stressor. Significant treatment effect of the herbal supplement versus placebo. ***p < 0.005.

A significant Treatment × Day interaction was observed for HR during the OMS (*F* (2, 207.35) = 5.76, *p* = 0.004). Here, a significant increase in HR was observed following the herbal supplement (73.29) as compared to placebo (70.93), following the acute dose only (*p* = 0.001) (Figure 14).

## Discussion

The present study investigated the acute and chronic effects of a herbal supplement containing *S. baicalensis*, *C. laevigata*, magnesium, and chromium on cognitive performance in both the presence and absence of a psychosocial stressor. Subjective measures of well-being, mood, and sleep were also assessed following interim (7 days) and chronic (15 days) supplementation.

Findings demonstrated that the herbal supplement led to a significant increase in the number of correct responses on a serial 7’s subtraction task as compared to placebo, overall and irrespective of acute or chronic dose. This finding was observed alongside a significant reduction in the number of errors on a serial 3’s subtraction task following an acute dose of the herbal supplement, as compared to placebo, when these tasks were performed in the presence of the OMS (1 hour post-intervention). In the absence of the stressor, the effects on cognitive performance were more variable. During the CDB, the herbal supplement led to a significant reduction in correct serial 3’s subtractions overall, relative to placebo. However, this finding was accompanied by a significant reduction in the number of RVIP false alarms on the third repetition, suggesting that the herbal supplement may have contributed to improved response accuracy over prolonged task engagement. Effects on individual task performance were also observed, with the herbal supplement leading to a significant reduction in reaction time during a picture recognition task, indicating enhanced processing speed. While numeric working memory reaction time increased following the herbal supplement, this did not translate into a decrease in accuracy, supporting that a speed-accuracy trade-off had not taken place.

In terms of the effects on well-being, mood, sleep, and anxiety, the herbal supplement led to a significant and pronounced reduction in general Trait anxiety, as compared to placebo overall and irrespective of dose, within 7 days. This was accompanied by a significant attenuation of total mood disturbance as rated by the POMS questionnaire, as well as a significant attenuation of feelings of anger-hostility following the herbal supplement, as compared to placebo, irrespective of dose. Fifteen days’ supplementation of the herbal supplement also led to a significant increase in total sleep time, but reductions in ratings of overall sleep quality were also observed irrespective of dose. During the psychosocial stressor, the herbal supplement led to a significant increase in HR as compared to placebo, following the acute dose only. There appeared to be no further positive or negative effects of treatment on outcome measures when an acute dose was superimposed on that of a chronic dose. The exploratory analysis of the PROMS showed that rates of social satisfaction increased, with significantly more participants taking the herbal supplement achieving ⩾30% increases in WHO-QOL social relationships scores at day 7. While the effect persisted at day 15, the difference from placebo was no longer significant.

Reflecting first on the cognitive effects, the herbal supplement significantly improved both serial 3’s and serial 7’s subtraction performance, however, this was only when these tasks were administered during the psychosocial stressor. During performance of one of the same tasks (serial 3’s) in the absence of the stressor, the herbal supplement led to significant decrements in performance. Interestingly, the positive treatment-related effects on cognition during the stressor were represented differently for each of the subtraction tasks; with an increase in number correct for the serial 7’s task and a reduction in number of errors for the serial 3’s task—a finding that may be explained by the different demands of the tasks themselves. Serial 7’s subtraction is a more difficult task, associated with increased cognitive demand due to the number of regrouping calculations required (Bristow et al., 2016). By contrast, the serial 3’s task primarily involves basic subtraction and is principally an attentional task. It is possible that in the current context, the positive effects observed as a result of treatment were focused on the more demanding aspects of each task (e.g. improving accuracy during serial 3’s subtractions and facilitating working memory during serial 7’s performance). Estimated mean data also showed a pattern of generally lower error rates in the serial 7’s and 17’s subtraction tasks during the stressor following the herbal supplement. However, given the lack of statistical confirmation (*p* > 0.1), this observation remains inconclusive and requires further investigation (see Supplemental File 5).

When these findings on cognition are considered in conjunction with the positive effects on Trait anxiety, one could reasonably suggest that the treatment is exerting a protective effect. So, during a stressful situation such as the OMS, the herbal supplement mitigates the effects of the stressor allowing for improved cognitive performance on tasks of working memory, executive function and attention. By contrast, the CDB is conducted in the absence of a stressor and is instead designed to be cognitively exhausting. Here, three tasks are repeated continuously over the course of 30 minutes (serials 3’s, serial 7’s and RVIP) resulting in a paradigm that has reliably been shown to induce mental fatigue (Kennedy et al., 2008, 2011; Reay et al., 2005, 2006). During the CDB in the current study, participants showed reduced accuracy in serial 3’s subtractions following the active treatment suggesting that the benefits of the supplement may be pronounced under acute stress but may not extend to all challenging cognitive settings such as the CDB. This highlights the potential relevance of situational factors—such as task duration, fatigue level, and stress intensity—in modulating the cognitive impact of the herbal supplement within the present population; those who self-report as experiencing relatively high levels of subjective stress. Indeed, evidence of this contextual effect has been demonstrated previously by our own lab. Here, a replication study failed to reproduce the cognitive effects that were observed in the original research when the stressor was omitted from the procedure (Dodd et al., 2024). As the anxiolytic and sedative properties associated with the active ingredients contained within the supplement likely underlie the aforementioned effects (Hui et al., 2002; Poleszak et al., 2004; Seo-Young and Hong-Sook, 2006; Walker et al., 2002), it would be reasonable to assume that they may interact with each of the contexts in-kind, rendering subjects more relaxed in their baseline state and better prepared to perform under elevated stress. In support of this, these positive effects were observed in the relative absence of other cognitive effects of treatment. For example, the pattern of effects was largely consistent in terms of the specific tasks concerned (serial subtractions) as well as the context in which they were seen (during the stressor as opposed to during the CDB).

When considering the outcomes from the other individual tasks, the pattern is less clear. Here, the herbal supplement led to a significant reduction in picture recognition reaction time, but also a significant increase in numeric working memory reaction time, as compared to placebo overall and irrespective of dose. Whilst these findings should not be dismissed, their presence amongst so many other cognitive tasks assessing reaction time (e.g. working memory and episodic memory), should be considered with caution.

Taken together, these findings suggest that the herbal supplement supports cognitive function in a nuanced manner, with benefits in specific tasks when experiencing periods of stress (psychosocial stress during the OMS) or conditions of sustained engagement (exhaustive conditions during CDB). The observed variability in effects across different cognitive domains indicates that the impact of the herbal supplement may be task-dependent, warranting further investigation into the contexts in which it is most effective.

In terms of previous research in this area, there are a limited number of studies that have looked at the effects of *S. baicalensis* and *C. laevigata* either alone or in combination with magnesium or chromium in humans. Few to none have looked at the effects upon cognition, stress or anxiety. Evidence from animal studies suggests a potential role of the flavonoids wogonin, oroxylin A and baicalin/baicalein, among the main chemical constituents of *S. baicalensis*. Wogonin has been shown to exert anxiolytic effects (Hui et al., 2002; Seo-Young and Hong-Sook, 2006), potentially through modulation of the GABA_A_ receptor via interaction at the benzodiazepine site (Hui et al., 2002). Baicalin and oroxylin A on the other hand, appear to interact with the dopaminergic system (Limanaqi et al., 2020; Yoon et al., 2013), potentially contributing to their anxiolytic and mood-enhancing effects, as demonstrated in pre-clinical studies (Limanaqi et al., 2020; Ma et al., 2024). Notably, baicalein/baicalin exhibits reasonable bioavailability (Limanaqi et al., 2020; Pang et al., 2016). *C. laevigata* contained within the formulation is sourced from the dried berries. Within the berries of *C. laevigata*, the major phenolic compounds are oligomeric procyanidins and their glycosides, primarily epicatechin but also catechin (Yang and Liu, 2012) and flavone compounds such as Rutin and Hyperosid (Nazhand et al., 2020). There is evidence from animal studies to suggest that in rodents epicatechin, rutin and hyperosid may mitigate anxiety-like behaviours (Can et al., 2010; Foudah et al., 2022; Kang et al., 2022; Stringer et al., 2015). Furthermore, a pilot study assessing the effects of 10 weeks’ administration with 500 mg hawthorn extract in humans, demonstrated a trend for reduced subjective anxiety. However, the effect appeared to be dependent on the specific formulation in which the hawthorn extract was administered (Walker et al., 2002). When administered in animals, magnesium has also shown anxiolytic-like activity, thought to be as a result of NMDA receptor inhibition (Młyniec et al., 2014; Poleszak et al., 2004). There is also some evidence to suggest an anxiolytic effect in humans (Boyle et al., 2017). Most notably in relation to cognition, chromium has been shown to improve memory function in older adults with early memory decline (Krikorian et al., 2010). The anxiolytic and mood-enhancing effects of the essential minerals and herbal extracts *S. baicalensis* and *C. laevigata* and their constituents may contribute to the observed improvements in cognitive performance under psychosocial stress in this study, given that positive mental states are linked to enhanced cognitive function (Dolcos, 2013; Pessoa, 2008).

Alongside the improvements observed in cognitive performance during the OMS stressor, there was also a pattern of increased HR following the herbal supplement (as compared to placebo), a finding which appeared to be driven by the effects on day 1 and occurred irrespective of the cognitive task the participant was completing (either serial 3’s, 7’s, 17’s). Indeed, at day 15, there appears to be little difference in HR between treatments. The reason for the increase in HR following the acute dose of the supplement is unclear, as the active constituents are not known to independently cause elevated HR. Consequently, the possibility of a synergistic effect between components cannot be ruled out. It is of note here, however, that there were no other treatment-related physiological effects of the OMS stressor (for example, no effects on salivary cortisol, salivary α-amylase or GSR). Previous research of *C. laevigata* has demonstrated that there is evidence to suggest anti-hypertensive and cardio-protective effects in humans. However, this appears to be dependent upon treatment length, with studies conducted over 12 weeks or more showing more consistent effects, and limited effects after acute dosing (Cloud et al., 2020; Degenring et al., 2003). It is also important to note that previous studies used higher doses of *C. laevigata* than the 80 mg daily dose administered here.

With regards to the subjective effects on well-being, mood and sleep, the pattern of reduced subjective general anxiety and improved ratings of mood overall following administration of the herbal supplement suggests that beneficial effects of treatment are apparent following ~7 days supplementation, at least in the present population (those who self-identify as experiencing stress). In keeping with the anticipated anxiolytic effects of treatment, the herbal supplement led to a significant reduction in general anxiety, as compared to placebo. Previous research suggests that both *Scutellaria* and *Crataegus* contained within the herbal supplement may possess anxiolytic effects. Whilst the majority of evidence for the herbal extracts is largely based on animal studies (Foudah et al., 2022; Hui et al., 2002; Kang et al., 2022; Lee et al., 2007; Ma et al., 2024; Seo-Young and Hong-Sook, 2006; Stringer et al., 2015), there is some evidence in humans (Walker et al., 2002) and this is notably for sustained effects on cognition over 30 days of supplementation (Yimam et al., 2016). Of the other active ingredients contained within the supplement, there is evidence to suggest that magnesium may be of benefit to those with a vulnerability to anxiety (Boyle et al., 2017), likely mediated via the NMDA-antagonistic and GABA-agonistic properties of magnesium (Murck and Steiger, 1998).

Interestingly, the present findings revealed a reduction in Trait but not State anxiety. Trait anxiety, being a more stable dispositional personality trait is generally considered less responsive than State anxiety to short-term interventions (Gidron, 2020), which makes this outcome notable. The observed pattern may also reflect methodological aspects such as the timing of the questionnaire’s administration: while the Trait questionnaire was completed at neutral timepoints (upon arrival and during the 6-week interim, at home), State anxiety was measured in close proximity to the OMS psychosocial stressor. This temporal context could have influenced State anxiety scores and potentially attenuated the visibility of any effects.

In relation to the effects on mood, a significant attenuation of total mood disturbance and a significant attenuation of feelings of anger-hostility were observed, irrespective of dose (overall). Interestingly and in line with these findings, there were also trends (*p* < 0.1) for reduced feelings of depression-dejection (*p* = 0.062); fatigue-inertia (*p* = 0.097) as well as tension-anxiety (*p* = 0.059) following the herbal supplement, as compared to placebo. Although the aforementioned findings failed to reach significance, in conjunction with the significant effects that are reported, they show a consistent pattern of improvement to mood after at least 7 days of supplementation with the herbal supplement (see Supplemental File 5). As previously discussed, there is very limited prior research looking at the effects of the extracts contained within the herbal supplement, either in isolation or in combination within humans, particularly in relation to mood/well-being. There is however evidence of reduction of depressive-like symptoms in animal models following *S. baicalensis* (Limanaqi et al., 2020; Zhu et al., 2006). This is thought to be as a result of the flavonoid baicalin, found in the root of the plant (from which the extract contained in the herbal supplement is sourced), and its ability to regulate the hypothalamic-pituitary-adrenal axis (Wang and Gao, 2023) and dopaminergic pathways (Limanaqi et al., 2020; Yoon et al., 2013). Anecdotal evidence to suggest a ‘calming effect’ of *C. laevigata* has also been demonstrated (Abascal and Yarnell, 2004), as well as evidence in animals and humans to suggest anti-depressant activity of chromium (Basiri et al., 2023; Młyniec et al., 2014). Clearly, however, more research is needed here.

Although total sleep time showed a clear increase after 15 days’ supplementation with the herbal supplement, changes in subjective assessments (e.g. psychological tension and overall sleep quality) were more complex. Notably, the mild decrease in psychological tension was driven primarily by changes in the placebo group, rather than by an increase in tension among individuals receiving the herbal supplement. Moreover, it should be noted that the absolute psychological tension ratings—ranging from 0.86 to 1.15 on a 0–4 scale—were nearly negligible. As for overall sleep quality, any potential improvement following the increase in longer sleep duration does not always translate directly into enhanced self-perceived quality, at least not in the short term. These observations highlight the multifaceted nature of sleep, suggesting that factors beyond mere duration may play a key role in shaping individuals’ subjective sleep experiences. For clarity, total sleep time is a measure of how long the participant spends sleeping as opposed to ‘time in bed’; which is a measure of how long a participant is in bed and could also include non-sleep-related activities. Previous research has indicated somnogenic effects of baicalin, the active constituent of *S. baicalensis* (Chang et al., 2011), with the *Crataegus* species also demonstrating some positive effects on sleep (Abbasi et al., 2021). This, in addition to being used traditionally as a sedative (Cui et al., 2023; Nazhand et al., 2020) may in part explain the effects on sleep seen here.

Despite the positive effects of treatment, there were limitations of the present study which should be acknowledged. The multi-ingredient nature of the intervention makes attribution of the effects of the supplement to any one of the active ingredients or their mechanisms difficult. Indeed, it may be that the active constituents work synergistically, however, since the ingredients were not tested individually it is not possible to determine this. A further limitation is the duration of the treatment period. Participants attended the lab following an acute dose and 15 days of treatment, with an interim assessment also completed at day 7 for the PROMS measures. Since most of the mood/well-being and cognitive effects occurred irrespective of dose, it would be interesting to determine if these findings could be maintained and/or more pronounced following a longer treatment period. Considering the positive effects on mood and anxiety, as well as cognition during the psychosocial stressor, future research would benefit from elucidating the effects of the herbal supplement following a longer treatment period to determine any additional benefits of treatment as well as optimum treatment duration. This was demonstrated with another supplement containing *S. baicalensis* root extract, for which a minimum 2-week intervention was required to achieve reasonable clinical effects on cognition (Yimam et al., 2016).

## Conclusion

The current study has demonstrated that 15 days supplementation with a formulation containing *S. baicalensis* and *C. laevigata* in combination with Magnesium and Chromium in a subjectively stressed population improves mood, alleviates subjective anxiety, and benefits cognitive performance during a psychosocial stressor. In the absence of a stressor, the effects on cognition are less consistent. Future research should explore longer treatment durations and clarify whether the observed effects apply solely to individuals with subjective stress or extend to the broader population. The adverse effects of stress are well established. This research has provided evidence for the mitigating effect of a herbal supplement containing magnesium and chromium on general mood/well-being and on cognition in the presence of a psychosocial stressor.

## Supplemental Material

sj-docx-1-jop-10.1177_02698811251381261 – Supplemental material for Effects of a Scutellaria baicalensis/Crataegus laevigata, magnesium and chromium supplement on stressed individuals: A randomised, double-blind, placebo-controlled, crossover trial Supplemental material, sj-docx-1-jop-10.1177_02698811251381261 for Effects of a Scutellaria baicalensis/Crataegus laevigata, magnesium and chromium supplement on stressed individuals: A randomised, double-blind, placebo-controlled, crossover trial by Fiona Dodd, Ramon Weishaupt, Philipp K. M. Katumba, Rian Elcoate and Emma Wightman in Journal of Psychopharmacology

sj-docx-2-jop-10.1177_02698811251381261 – Supplemental material for Effects of a Scutellaria baicalensis/Crataegus laevigata, magnesium and chromium supplement on stressed individuals: A randomised, double-blind, placebo-controlled, crossover trial Supplemental material, sj-docx-2-jop-10.1177_02698811251381261 for Effects of a Scutellaria baicalensis/Crataegus laevigata, magnesium and chromium supplement on stressed individuals: A randomised, double-blind, placebo-controlled, crossover trial by Fiona Dodd, Ramon Weishaupt, Philipp K. M. Katumba, Rian Elcoate and Emma Wightman in Journal of Psychopharmacology

sj-docx-3-jop-10.1177_02698811251381261 – Supplemental material for Effects of a Scutellaria baicalensis/Crataegus laevigata, magnesium and chromium supplement on stressed individuals: A randomised, double-blind, placebo-controlled, crossover trial Supplemental material, sj-docx-3-jop-10.1177_02698811251381261 for Effects of a Scutellaria baicalensis/Crataegus laevigata, magnesium and chromium supplement on stressed individuals: A randomised, double-blind, placebo-controlled, crossover trial by Fiona Dodd, Ramon Weishaupt, Philipp K. M. Katumba, Rian Elcoate and Emma Wightman in Journal of Psychopharmacology

sj-docx-4-jop-10.1177_02698811251381261 – Supplemental material for Effects of a Scutellaria baicalensis/Crataegus laevigata, magnesium and chromium supplement on stressed individuals: A randomised, double-blind, placebo-controlled, crossover trial Supplemental material, sj-docx-4-jop-10.1177_02698811251381261 for Effects of a Scutellaria baicalensis/Crataegus laevigata, magnesium and chromium supplement on stressed individuals: A randomised, double-blind, placebo-controlled, crossover trial by Fiona Dodd, Ramon Weishaupt, Philipp K. M. Katumba, Rian Elcoate and Emma Wightman in Journal of Psychopharmacology

sj-xlsx-5-jop-10.1177_02698811251381261 – Supplemental material for Effects of a Scutellaria baicalensis/Crataegus laevigata, magnesium and chromium supplement on stressed individuals: A randomised, double-blind, placebo-controlled, crossover trial Supplemental material, sj-xlsx-5-jop-10.1177_02698811251381261 for Effects of a Scutellaria baicalensis/Crataegus laevigata, magnesium and chromium supplement on stressed individuals: A randomised, double-blind, placebo-controlled, crossover trial by Fiona Dodd, Ramon Weishaupt, Philipp K. M. Katumba, Rian Elcoate and Emma Wightman in Journal of Psychopharmacology

sj-xlsx-6-jop-10.1177_02698811251381261 – Supplemental material for Effects of a Scutellaria baicalensis/Crataegus laevigata, magnesium and chromium supplement on stressed individuals: A randomised, double-blind, placebo-controlled, crossover trial Supplemental material, sj-xlsx-6-jop-10.1177_02698811251381261 for Effects of a Scutellaria baicalensis/Crataegus laevigata, magnesium and chromium supplement on stressed individuals: A randomised, double-blind, placebo-controlled, crossover trial by Fiona Dodd, Ramon Weishaupt, Philipp K. M. Katumba, Rian Elcoate and Emma Wightman in Journal of Psychopharmacology
