# Supplementary material for: Effects of a Scutellaria baicalensis/Crataegus laevigata, magnesium and chromium supplement on stressed individuals: A randomised, double-blind, placebo-controlled, crossover trial
Source: J Psychopharmacol. 2025 Nov 5;39(12):1420–36. doi: 10.1177/02698811251381261 (PMC12672942; doi:10.1177/02698811251381261)
Supplement: sj-docx-1-jop-10.1177_02698811251381261 – Supplemental material for Effects of a Scutellaria baicalensis/Crataegus laevigata, magnesium and chromium supplement on stressed individuals: A randomised, double-blind, placebo-controlled, crossover trial [file sj-docx-1-jop-10.1177_02698811251381261.docx]

**Supplemental File 1 – Eligibility Criteria**

Participants were eligible to participate if they:

- Self-identified as experiencing stress
- Self-reported a score of ≥ 13 on the Perceived Stress Scale (PSS)
- Self-reported as being in good health
- Were aged between 18 and 75 years of age

Participants were not eligible to participate if they:

- Scored 12 or less on the pre-screen Perceived Stress Scale
- Reported any pre-existing medical condition/illness which would impact taking part in the study (exceptions to this included controlled hyper/hypothyroidism, hay fever, high cholesterol, and reflux related conditions)
- Reported currently taking prescription medications (explicit exceptions to this were contraceptive treatments for female participants, thyroid medications, topical skin treatments, those used in the treatment of high cholesterol, and reflux-related conditions, and those taken “as needed” in the treatment of asthma and hayfever)
- Were assessed as having high blood pressure (systolic over 159 mm Hg or diastolic over 99 mm Hg), as measured within the lab
- Were assessed as having a Body Mass Index (BMI) outside of the range 18.5-35 kg/m^2^
- Were pregnant, seeking to become pregnant or lactating
- Reported learning and/or behavioural difficulties such as dyslexia or ADHD
- Reported a visual impairment that could not be corrected with glasses or contact lenses (including colour-blindness)
- Smoked tobacco or vaped nicotine or used nicotine replacement products (if they had recently quit smoking or using replacements they must have stopped using them altogether for a period of 3 months before participating in the study)
- Reported excessive caffeine intake (>500 mg per day)
- Reported relevant food allergies/ intolerances/ sensitivities
- Had taken antibiotics within the past 4 weeks
- Had taken dietary supplements e.g. vitamins, omega 3 fish oils etc. in the last 4 weeks
- Reported any health condition that would prevent fulfilment of the study requirements (this included non-diagnosed conditions for which no medication may be taken)
- Were unable to complete all of the study assessments
- Were participating in other clinical or nutrition intervention studies, or had done so in the 4 weeks prior to participation
- Had been diagnosed with/ undergoing treatment for alcohol or drug abuse in the last 12 months
- Had been diagnosed with/ undergoing treatment for a psychiatric disorder in the last 12 months, including a medical diagnosis of anxiety or depression.
- Reported suffering frequent migraines that require medication (more than or equal to 1 per month)
- Reported having oral disease
- Reported any known active infections
- Did not have a bank account (required for payment)
- Were likely to be non-compliant with regards treatment consumption
