# Supplementary material for: Effects of a Scutellaria baicalensis/Crataegus laevigata, magnesium and chromium supplement on stressed individuals: A randomised, double-blind, placebo-controlled, crossover trial
Source: J Psychopharmacol. 2025 Nov 5;39(12):1420–36. doi: 10.1177/02698811251381261 (PMC12672942; doi:10.1177/02698811251381261)
Supplement: sj-docx-2-jop-10.1177_02698811251381261 – Supplemental material for Effects of a Scutellaria baicalensis/Crataegus laevigata, magnesium and chromium supplement on stressed individuals: A randomised, double-blind, placebo-controlled, crossover trial [file sj-docx-2-jop-10.1177_02698811251381261.docx]

**Supplemental File 2 – Treatment Composition**

**Active Intervention Herbal Supplement**

The active treatment contained per tablet: *Scutellaria baicalensis* root extract (400mg) and *Crataegus laevigata fruit extract* (40mg), as well as xylitol, magnesium citrate (366 mg corresponding to 56.3 mg Mg), mannitol, magnesium salts of edible fatty acids, gum tragacanth, malic acid, natural lemon and blood orange flavouring, steviol glycosides from stevia, and chromium chloride (107 µg corresponding to 20 µg Cr).

**Matched Placebo**

The placebo treatment contained microcrystalline cellulose, xylitol, mannitol, stearic acid, gum tragacanth, malic acid, natural lemon and blood orange flavouring and steviol glycosides from stevia but did not contain the active ingredients.
