# Supplementary material for: Effects of a Scutellaria baicalensis/Crataegus laevigata, magnesium and chromium supplement on stressed individuals: A randomised, double-blind, placebo-controlled, crossover trial
Source: J Psychopharmacol. 2025 Nov 5;39(12):1420–36. doi: 10.1177/02698811251381261 (PMC12672942; doi:10.1177/02698811251381261)
Supplement: sj-docx-3-jop-10.1177_02698811251381261 – Supplemental material for Effects of a Scutellaria baicalensis/Crataegus laevigata, magnesium and chromium supplement on stressed individuals: A randomised, double-blind, placebo-controlled, crossover trial [file sj-docx-3-jop-10.1177_02698811251381261.docx]

**Supplemental file 3 – Full description of all COMPASS tasks**

**Individual Cognitive Tasks**

*Numeric Working Memory task.* Five single digits were presented sequentially for the participant to hold in memory. Thirty probe digits (15 targets and 15 distractors) appeared on screen and the participant made a yes/no response. The task was repeated three times. Outcomes: Speed (measured in reaction time (RT), with a lower score indicating better performance) and accuracy of performance (measured as a percentage, with a higher score indicating better performance).

*Choice Reaction Time.* Participants responded as quickly as possible to a stimulus (upwards pointing arrow). Outcome: Speed of response (measured in RT, with a lower score indicating better performance).

*Corsi Blocks.* Nine blue squares on a black background were displayed on the screen. Some of the blue squares changed to red and back to blue again in a sequence. Participants were required to remember this sequence. The task was repeated five times at each level of difficulty with the sequence span increasing from 4 upwards, until the participant could no longer correctly recall the sequences. A higher score indicating better performance.

*Peg and Ball.* Participants moved coloured balls on pegs using the mouse and cursor to match a target display. Difficulty increased from problems requiring 3 moves, to 4 moves and 5 moves. Outcomes: Total errors (measured as a number with a lower score indicating better performance), thinking time and completion time (measured in RT, with a lower score indicating better performance.

*Immediate and Delayed Word Recall.* Fifteen words were presented one at a time during the stimulus presentation period and participants were required to write down as many of the 15 presented words that they could remember immediately after this period and also during the delayed recall/recognition period. Outcome: Accuracy during each phase (measured by number correct, with a higher score indicating better performance and number of errors, with a lower score indicating better performance).

*Name to Face Recall.* Twelve faces with first and last names were presented one at a time during the stimulus presentation period. During the delayed recall/recognition period participants were presented with the faces and were asked to recall the name paired with each face by choosing from four first and four last names. Outcomes: Accuracy (measured as a percentage, with a higher score indicating better performance) and speed of response (measured in RT, with a lower score indicating better performance).

*Delayed Word Recognition.* Participants responded yes/no to 15 words that had been presented during the stimulus presentation period, plus 15 novel distractor words. Outcomes: Accuracy (measured as a percentage, with a higher score indicating better performance) and speed of response (measured in RT, with a lower score indicating better performance).

*Picture Recognition.* Fifteen pictures were presented one at a time during the stimulus presentation period. During the delayed recall/recognition period the same 15 pictures, plus 15 distractor pictures were presented, with participants making a yes/no response indicating whether the picture was in the original set. Outcomes: Accuracy (measured as a percentage, with a higher score indicating better performance) and speed of response (measured in RT, with a lower score indicating better performance).

**Cognitive Demand Battery**

The objective of this battery was to assess the impact of treatment on speed/accuracy and mental fatigue during continuous performance of cognitively demanding tasks. Here participants completed the ~10-minute battery of tasks (Serial 3s (2 minutes), Serial 7s (2 minutes), RVIP (5 minutes) and a mental fatigue scale (~ 1 minute)), 3 times in immediate succession (i.e. for a continuous period of 30 minutes). Application of this battery has been shown to reliably increase self-ratings of ‘mental fatigue’ and to be sensitive to a number of herbal and natural interventions (Kennedy et al., 2008; Kennedy & Scholey, 2004; Reay, Kennedy, & Scholey, 2005, 2006a, 2006b). Details of the 10-minute battery are given below:

*Serial threes subtraction task (2 mins):* Computerised versions of the serial subtraction tasks were implemented using tests of 2-minute duration. Participants were required to count backwards in threes from a given number as quickly and as accurately as possible using the number keys to enter each response. A random starting number between 800 and 999 was presented on the computer screen, which was cleared by the entry of the first response. The task was scored for number of correct responses (with a higher score indicating better performance) and number of errors (with a lower score indicating better performance). In the case of incorrect responses subsequent responses were scored as positive if they were scored as correct in relation to the new number.

*Serial sevens subtraction task (2 mins):* This was identical to the serial threes task with the exception that it involved the serial subtraction of sevens.

*Rapid Visual Information Processing task (RVIP-5 mins):* The participant was required to monitor a continuous series of digits for targets of three consecutive odd or three consecutive even digits. The digits were presented at the rate of 100 per minute and the participant responded to the detection of a target string by pressing the response button as quickly as possible. The task was continuous and lasted for 5 minutes, with 8 correct target strings being presented in each minute. The task was scored for percentage of target strings correctly detected (with a higher score indicating better performance), average reaction time for correct detections (with a lower score indicating better performance), and number of false alarms (with a lower score indicating better performance).

*‘Mental fatigue’ visual analogue scale:* Participants rated their subjective ‘mental fatigue’ state by making a mark on a 100 mm line with the end points labelled ‘not at all’ (left hand end) and ‘very much so’ (right hand end). On this scale 0 indicated not at all mentally fatigued and 100 indicated very much mentally fatigued.
