# Supplementary material for: Effects of a Scutellaria baicalensis/Crataegus laevigata, magnesium and chromium supplement on stressed individuals: A randomised, double-blind, placebo-controlled, crossover trial
Source: J Psychopharmacol. 2025 Nov 5;39(12):1420–36. doi: 10.1177/02698811251381261 (PMC12672942; doi:10.1177/02698811251381261)
Supplement: sj-docx-4-jop-10.1177_02698811251381261 – Supplemental material for Effects of a Scutellaria baicalensis/Crataegus laevigata, magnesium and chromium supplement on stressed individuals: A randomised, double-blind, placebo-controlled, crossover trial [file sj-docx-4-jop-10.1177_02698811251381261.docx]

**Supplemental file 4 – Full procedure**

Participants initially completed a telephone pre-screen where they provided consent to participate and confirmed they met the study eligibility criteria. They then attended the Brain, Performance, Nutrition Research Centre laboratory (Northumbria University, UK) on five separate occasions. The first was an in-person screening and training visit where in-person informed written consent was obtained and demographic and anthropometric measurements (height, weight and blood pressure (BP)) were taken. Participants were also trained on the cognitive tasks they would be required to complete. Following this visit participants attended the laboratory at a pre-arranged time in the morning between 10.00 am and 11.00 am on four separate occasions (visits 1 – 4). The first and third visits comprised the baseline assessments. Visits 2 and 4 were chronic assessments and occurred 15 days (+/- 2 days) after visits 1 and 3, respectively. Each visit was identical, except for the intervention consumed at and in between visits 1 and 2 and visits 3 and 4. See **Figure 3** for a schematic depicting the timeline of study.

Prior to each testing visit, participants were asked to avoid alcohol and the intake of analgesic and other over the counter (OTC) medication for 24 hours and from systemic antihistamines for 48 hours. They were allowed to consume their normal breakfast on the day of testing but were asked to avoid all beverages other than water from waking and to abstain from any caffeine containing food and beverages including decaffeinated varieties. All food had to be finished at least 90 minutes prior to the session, and participants were instructed that the food consumed prior to testing visit 1 was to be replicated prior to all testing visits (a diary was used to record this). Upon arrival at visits 1 – 4, participants were screen for continued eligibility criteria and any changes to medications or health were reported. Following screening, participants provided baseline GSR and HR readings (~5 minutes), a day-baseline saliva sample and the PROMS questionnaires (CSD, PROMIS-SD, PSS, STAI-T, DASS-21, WHOQOL-Bref, POMS) to assess psychological mood, wellbeing and sleep. Following this, participants completed a baseline assessment of the COMPASS cognitive task battery (see **Figure 1** for a schematic depicting the individual COMPASS tasks). After a short (~15 minute) break, participants were taken to an ‘interview’ room where they underwent a baseline completion of the OMS for 20 minutes in front of a panel of two observers whilst also being video recorded and having their GSR and HR readings measured throughout. The STAI-State was completed and a saliva sample was provided in the lab immediately prior to and after the OMS. Participants were then provided with their daily treatment (2 x tablets; bolus dose), followed by a standardised lunch of a white-bread cheese sandwich, packet of ready salted crisps and a custard pot. The second (post-dose) assessment started after a 60 minute long absorption period and here participants repeated the COMPASS cognitive assessment followed by the OMS (see **Figure 2** for a schematic depicting the procedure during testing visits 1 – 4). Before leaving on visits 1 and 3 participants were provided with their treatment (14 days’ worth of treatment +/- additional doses in case of delayed return for the chronic visit) and their treatment diary which was used to record the date and time treatment was taken and any illness/medication required over the treatment period. Starting the next day, participants were instructed to take one tablet in the morning (at least an hour after breakfast) and one in the afternoon (at least one hour before the evening meal) and were advised to chew the tablets and take them with water. If participants forgot to take a dose, they were advised to take the dose as soon as they remembered and continue with the dosing schedule as normal. If a dose was missed completely then participants were simply advised to make a note in their diary. Missed doses were not replaced.

At visits 2 and 4 participants returned any unused treatment and their treatment diaries and were instructed not to consume any treatment at home prior to attending their session (as treatment would be consumed in the laboratory, as per their first visit). A minimum 14-day washout period (with a maximum of 28 days) was observed between each treatment period. At the end of visit 4 participants completed a treatment guess, were debriefed and remunerated for their time. The testing visits lasted approximatley 4 hours 30 minutes. In order to assess the interm effects of treatment on mood, wellbeing and sleep outcomes, participants were also instructed to complete a PROMS assessment at home on day 8 (+/-2) within each treatment period.
